# Translocations as Experiments in the Ecological Resilience of an Asocial Mega-Herbivore

**DOI:** 10.1371/journal.pone.0030664

**Published:** 2012-01-25

**Authors:** Wayne L. Linklater, Jay V. Gedir, Peter R. Law, Ron R. Swaisgood, Keryn Adcock, Pierre du Preez, Michael H. Knight, Graham I. H. Kerley

**Affiliations:** 1 Centre for Biodiversity and Restoration Ecology, School of Biological Sciences, Victoria University of Wellington, Wellington, New Zealand; 2 Department of Zoology, Centre for African Conservation Ecology, Nelson Mandela Metropolitan University, Port Elizabeth, South Africa; 3 PRLDB Modeling, Monroe, New York, United States of America; 4 San Diego Zoo Global Institute for Conservation Research, Escondido, California, United States of America; 5 IUCN African Rhino Specialist Group, Hilton, KwaZulu-Natal, South Africa; 6 Directorate of Scientific Services, Ministry of Environment and Tourism, Windhoek, Namibia; 7 Park Planning and Development, South African National Parks, Port Elizabeth, South Africa; University of Pretoria, South Africa

## Abstract

Species translocations are remarkable experiments in evolutionary ecology, and increasingly critical to biodiversity conservation. Elaborate socio-ecological hypotheses for translocation success, based on theoretical fitness relationships, are untested and lead to complex uncertainty rather than parsimonious solutions. We used an extraordinary 89 reintroduction and 102 restocking events releasing 682 black rhinoceros (*Diceros bicornis*) to 81 reserves in southern Africa (1981–2005) to test the influence of interacting socio-ecological and individual characters on post-release survival. We predicted that the socio-ecological context should feature more prominently after restocking than reintroduction because released rhinoceros interact with resident conspecifics. Instead, an interaction between release cohort size and habitat quality explained reintroduction success but only individuals' ages explained restocking outcomes. Achieving translocation success for many species may not be as complicated as theory suggests. Black rhino, and similarly asocial generalist herbivores without substantial predators, are likely to be resilient to ecological challenges and robust candidates for crisis management in a changing world.

## Introduction

Translocations, or movement of species between habitats, are remarkable experimental tests of the evolutionary capacity of species [1] and our ecological understanding [2]. Translocation success, or failure, at individual and population scales should be predicted by theoretical relationships between demographic and socio-ecological characteristics, and evolutionary fitness [3], [4]. Translocations for reintroduction and restocking to restore and manage populations are also key to species rescue and recovery [5] and rapid progress demands that we find parsimonious guidelines for success [6]. The use of translocations as a conservation tool is expected to increase [7] due to the growing ranks of conservation-reliant species [8] and requirement for assisted migration [9] with climate change induced range shifts for many species [10]. With the need for this interventionist strategy on the rise, managers cannot afford to be unnecessarily timid or waste resources testing translocation strategies that bring only small, incremental improvements. General principles from evolutionary ecology that can be applied widely in the design of translocation programs are required.

Translocation success rates are generally poor [3], [4], [11], [12]. The large number of elaborate hypotheses for translocation success and potential for important interactions amongst variables has led to complex uncertainty rather than simple solutions. The datasets required to test hypotheses for translocation success are also complex hierarchies of information because individuals may be released as groups to sites which may receive multiple releases over time. The datasets required to test such multi-level, nested hypotheses are necessarily large but rarely available and so most hypotheses for many species have not been tested. Further, hierarchical data and multivariate hypotheses cannot be treated using conventional correlation and regression [13], [14] in the way that most hypotheses have been tested [3], [4], [11], [12]. Consequently, current best-practice in the translocation of wildlife is based largely on anecdote or, at best, relationships that might be spurious, commensurate with under- and over-fitting of multivariate data [15], [16]. The utility of general evolutionary ecological principles has not been tested.

The critically endangered black rhinoceros (*Diceros bicornis*) has an extraordinary documented history of translocations [17], [18]. From our previous analyses of their post-release survival, we have drawn conclusions and made recommendations, particularly about the importance of an individuals' age over demographic or habitat influences (e.g., cohort size, population density and habitat quality) when restocking [19]. In comparison, analyses of reintroductions have not revealed strong influences on success. Outcomes were ambiguous, although the poorest habitats and small cohorts including only bulls or large cohorts including several mothers with dependent calves were weakly associated with greater mortality of bulls and calves, respectively, during the first year [19]. Thus, previous work has downplayed the role of socio-ecological influences on translocation success, although it does not address the potential complexity of influences on survival stemming from interactions amongst variables. Some variables, although not influential on their own, may nevertheless have a synergistic effect when they interact with other variables. In particular, the absence of interactions in previous analysis may explain why reintroduction success was so poorly explained and why ecological and demographic influences appeared unimportant to restocking success [19].

After restocking events we expect the socio-ecological context to be more complex and influential due to conflict when newly released individuals encounter established residents already occupying the better habitats. We expect residents to have a ‘home advantage’ during aggressive confrontations and post-release competition for habitat and mates. In reintroductions, where releases occur in areas that no longer support a resident population, social conflict and competition are likely to be less important. Thus, interactions amongst supported variables might improve the predictive power of models designed to improve post-release survival. Our objective here is to advance the understanding of establishment success after reintroduction and restocking by explicitly modeling interactions amongst socio-ecological, demographic and individual rhinoceros characters. To this end we applied an extraordinary record of 682 black rhinoceros released into 81 reserves in Namibia and South Africa over 25 years (1981–2005) to test hypotheses for establishment success (i.e., survival to one year post-release) after 89 reintroduction and 102 restocking events.

## Results

**Figure 1:**
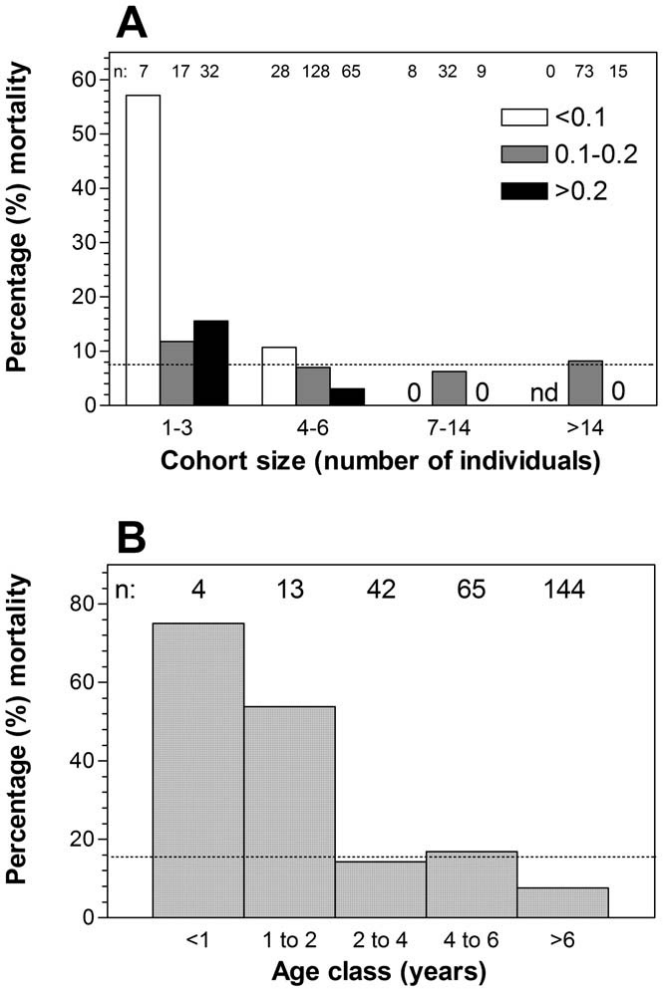
Post-release mortality in black rhinoceros (*Diceros bicornis*) after (A) reintroduction and (B) restocking. Cohort size and habitat quality (estimated carrying capacity <0.1, 0.1–0.2 or >0.2 rhino per km^2^) explained reintroduction mortality while age class explained deaths after restocking. Age classes conform to Hitchins' A (calf) to F (adult) aging scheme [31]. Numbers of rhino (i.e., n) in each category are indicated above each bar. nd = no data. The dash line across each indicates mean mortality rate for all reintroduction (A) and restocking (B) events.

The model describing the interaction between cohort size and habitat quality performed the best and improved substantially on previous leading models for reintroduction success (Table 1). Models including the interaction between cohort size and habitat quality contributed 92.5% of Akaike weights and were the only models to out-perform the base model without fixed-effects (Table 1). A simple, positive interaction between cohort size and habitat quality was supported (Fig. 1A) and substantially exceeded the explanatory power of two influences previously identified as potentially important for reintroduction success: the proportion of young and bulls in release cohorts which received no support (ΔAIC_c_>10, ω<0.004, Table 1).

**Table 1:** Results summary of the 29 candidate models for reintroduction mortality risk among 89 cohorts and 414 reintroduced black rhino.

Model/Hypothesis	*K*	AIC_c_	ΔAIC_c_	ω*_i_*
Cohort size * Habitat quality (Wolf et al. 1998)	5	222.8	0.0	0.635
Cohort size * Habitat quality * Proportion bulls in cohort	8	224.6	1.7	0.270
Cohort size * Habitat quality * Proportion mothers with calves in cohort	8	229.8	6.9	0.020
**Base model (including only the random effect for reserve)**	**2**	**231.1**	**8.3**	**0.010**
Cohort size * Proportion mothers with calves in cohort	5	231.2	8.4	0.010
Habitat quality	3	232.5	9.6	0.005
*Post-release adult density (Linklater & Swaisgood 2008, Hitchins & Anderson 1983)*	*3*	*232.6*	*9.7*	*0.005*
*Cohort size (Fischer & Lindenmayer 2000)*	*3*	*232.6*	*9.7*	*0.005*
Proportion bulls in cohort * Habitat quality	5	232.6	9.8	0.005
*Proportion bulls in cohort*	*3*	*233.0*	*10.1*	*0.004*
*Cohort size+Habitat quality (Griffith et al. 1989)*	*4*	*233.6*	*10.8*	*0.003*
Age	7	233.7	10.8	0.003
*Proportion mothers with calves in cohort*	*3*	*233.9*	*11.0*	*0.003*
*Number of mothers with calves+Proportion mothers with calves in cohort*	*4*	*233.9*	*11.1*	*0.003*
Cohort size * Post-release adult density	5	233.9	11.1	0.003
Cohort adult sex ratio * Sex	5	234.1	11.3	0.002
*Reserve size+Post-release adult density (Linklater & Swaisgood 2008b)*	*4*	*234.4*	*11.6*	*0.002*
*Number of bulls+Proportion of bulls in cohort*	*4*	*234.5*	*11.7*	*0.002*
*Source-recipient reserve size difference+Previously translocated*	*4*	*234.6*	*11.8*	*0.002*
*No. sub-adults+Proportion of cohort that are sub-adults*	*4*	*234.7*	*11.9*	*0.002*
*Source-recipient habitat quality difference+Translocation distance*	*4*	*235.0*	*12.2*	*0.002*
Post-release adult density * Proportion of carrying capacity occupied	5	235.5	12.7	0.001
Proportion of mothers with calves * Habitat quality	5	235.5	12.7	0.001
No. bulls * Proportion bulls	5	235.7	12.9	0.001
Reserve size * Post-release adult density	5	235.8	13.0	0.001
Cohort size * No. bulls	5	235.9	13.1	0.001
Cohort size * Proportion bulls	5	235.9	13.1	0.001
*Age+Cohort size+Habitat quality (Brett 1998)*	*9*	*236.6*	*13.8*	*0.001*
*Age+Reserve size+Post-release adult density (Walker 1994)*	*9*	*237.2*	*14.4*	*0.001*

Models are in descending order from most to least supported based on Akaike second-order Information Criteria (AIC_c_). Leading models from previous analyses without interaction terms [*9]*, [23**] are italicized. The model without fixed effects is indicated in bold type. A ‘*’ indicates an interaction term in the regression between two variables and, by implication, predictors in interactions were also present additively in models (e.g., a*b refers to model including a+b+a*b as fixed effects).

In contrast and unexpectedly, interactions of cohort and site-level variables representing population demography and habitat did not improve upon the restocking model including only age (Table 2) and so confirmed previous conclusions about the vulnerability of younger individuals when supplementing existing populations (Fig. 1B). Young are much less vulnerable after reintroduction (Age model: ΔAIC_c_>10, ω<0.003, Table 1), a finding consistent with our prediction that the risks inherent in translocation to restock carry disproportionately higher risks for young rhinoceros. Importantly however, and contrary to predictions, the vulnerability of young after restocking is sufficient to account for variation in establishment success without recourse to complex interactions with demographic and ecological characters. Models including age class contribute 100.0% of Akaike weights and were the only models to out-perform a model without fixed-effects (Table 2).

**Table 2:** Results summary of the 23 candidate models for restocking mortality risk among 102 cohorts of black rhino released into 48 reserves and including 273 individuals.

Model/Hypothesis	*K*	AIC_c_	ΔAIC_c_	ω*_i_*
*Age*	*7*	*203.5*	*0.0*	*0.321*
Age+Post-release adult sex ratio	8	204.4	0.8	0.212
*Age+Cohort adult sex ratio*	*8*	*205.2*	*1.6*	*0.142*
*Age+Sex*	*8*	*205.5*	*1.9*	*0.123*
*Age+Resident bull density (Adcock et al. 1998)*	*8*	*205.7*	*2.1*	*0.111*
*Age+Reserve area+Post-release adult density (Walker 1994)*	*9*	*207.8*	*4.3*	*0.038*
*Age+Cohort size+Resident adult density+Habitat quality (Brett 1998)*	*10*	*208.5*	*4.9*	*0.027*
*Age+Sex+Cohort size+Post-release adult density+Habitat quality (Brett 1998)*	*11*	*208.6*	*5.1*	*0.025*
**Base model (including only the nested random effect for cohort and reserve)**	**3**	**219.4**	**15.9**	**0.000**
Resident adult density * Habitat quality	6	223.2	19.7	0.000
Resident bull density * Habitat quality	6	224.6	21.1	0.000
Cohort size * Habitat quality	6	224.7	21.2	0.000
Habitat quality * Proportion of carrying capacity occupied	6	225.2	21.7	0.000
Post-release adult density * Habitat quality	6	225.2	21.7	0.000
Post-release adult sex ratio * Sex	6	221.9	18.4	0.000
Post-release adult sex ratio * Habitat quality	6	223.5	20.0	0.000
Cohort size * Final adult density	6	225.4	21.9	0.000
Cohort size * Resident adult male density	6	225.4	21.9	0.000
Cohort size * Resident adult density	6	225.2	21.7	0.000
Post-release adult sex ratio * Post-release adult density	6	223.9	20.4	0.000
Post-release adult density * Sex	6	222.8	19.3	0.000
Resident adult density * Sex	6	222.9	19.4	0.000
Resident adult male density * Sex	6	222.0	18.5	0.000
Resident adult density * Habitat quality	6	223.2	19.7	0.000
Resident adult male density * Habitat quality	6	224.6	21.1	0.000
Habitat quality * Sex	6	224.2	20.7	0.000

Models are in descending order from most to least supported based on Akaike second-order Information Criteria (AIC_c_). Leading models from previous analyses without interaction terms [*19]*, [23**] are italicized. The model without fixed effects is indicated in bold type. A ‘*’ indicates an interaction term in the regression between the two variables and, by implication, predictors in interactions were also present additively in models (e.g., a*b refers to model including a+b+a*b as fixed effects).

Lastly, support for base models (including only random effects for introduction and reserve) would be evidence that important hypotheses and predictors were not represented by the current analysis. That the base models instead received such poor support (reintroduction: ΔAIC_c_ = 8.3, ω = 0.010; restocking: ΔAIC_c_ = 15.9, ω = 0.000) gives us confidence in the value of the leading models to explain and predict variation in the post-release survival of black rhinoceros amongst cohorts and sites.

## Discussion

Information-Theoretic analyses deliver parsimony. An indication, therefore, of the extraordinary power of age to explain restocking success is that it contributed a large number of parameters to leading models (age is represented by four classes defining the first six years and an adult class) that performed better than smaller models. The risks posed to younger black rhinoceros when restocking are probably numerous and diverse (e.g., social asymmetries of competition and conflict, inter-specific conflict, resource unfamiliarity, disease, misadventure) such that no single risk dominates. While the addition of cohort and post-release adult sex ratio, sex, resident and post-release densities, cohort size, and habitat quality with age were also ranked highly, their explanatory power was not sufficient, at least for the dataset considered, to warrant any other action than avoiding the use of sub-adults, especially calves, when restocking black rhinoceros.

The interaction between cohort size and habitat quality resulted in extraordinarily high mortality rates after reintroductions of the smallest cohorts to habitat with the lowest carrying capacities. Large improvements in reintroduction success might be achieved by avoiding release of cohorts with fewer than four individuals, especially into poor quality habitat. Where reintroductions to poorer quality habitats are required, cohorts larger than six should be favored. The reason for the extraordinarily high mortality rates amongst individuals from small cohorts reintroduced to the poorest habitats is unclear. Perhaps normative social relationships amongst peers are important [20]? Even in the relatively asocial black rhinoceros, peers may help individuals refine habitat and food choices, especially in marginal habitat where resources are more heterogeneous in time and space. Normative behavior and conspecific attraction may facilitate habitat discovery and learning in novel environments. It is also possible that unsatisfied mate-choice or social behaviors encourage long-distance movements or displace maintenance behaviors after release such that individuals acclimate poorly in the absence of suitable mates or friends [21]. Small release cohorts might not provide the necessary peers or mates for successful post-release adjustment.

Mortality risks differ between reintroduction and restocking but model outcomes share similarities in those that are not supported, especially those describing interactions between individual characters (age, sex, experience) and metrics of population density and habitat quality. Thus, complex models representing themes of resource availability and competition and their interaction with individual characteristics continue to be unsupported [19]. Although authors have recommended large release cohorts [3], [4], [11], [18] including individuals that are not predator-, competitor-, or translocation-naïve [22], [23], and favoring large reserves with low conspecific density [24] and high-quality [3], [4], [18] or familiar [25] habitat, only two of these factors and their interaction were important for black rhinoceros, and only when reintroducing populations. The lack of support for the more complex models of translocation success, at least for black rhinoceros, indicates that the role of ecological and demographic influences is weaker than previously thought. Indeed, many relationships identified previously from simple correlations or regressions are probably spurious. This analysis confirms that importance has been mistakenly attributed to complexes of ecological and demographic influences, albeit for strong theoretical reasons, that are instead more simply explained.

Why do black rhinoceros in southern Africa defy the expectations of adaptive theory for important relationships between socio-ecological characters and metrics of fitness like survival after translocation? Such a finding appears to contradict our knowledge about the intensity of intra- and inter-specific competition and conflict, and habitat preferences amongst rhinoceros. Certainly, the successful translocation of other species appears to be demographically and socio-ecologically complex. Such species, however, are either more selective herbivores requiring smaller amounts of higher quality browse or grass, cooperative or gregarious breeders, predators whose prey (cf. browse and grass) is elusive, or they are prey of sympatric predators [22], [26], [27], [28]. Mega-herbivores appear to escape the constraints of predators that perhaps cause the failure of other ungulate introductions [29]. The simplicity of rules for black rhinoceros translocation, therefore, might be unusual, or at least confined to other similarly large, generalist herbivores with wide biogeographic ranges and largely asocial habits.

Importantly, the degree to which black rhinoceros are robust to a major life-history event like translocation, even into resident populations (i.e., restocking), raises important implications for the understanding of their ecology and conservation, especially in a changing climate. Species vary in their adaptive capacity for ecological change and their resiliency to drastic types of management like assisted migration [1]. Experience translocating black rhinoceros leads us to expect their populations to be resilient to ecological challenges like climate change compared to other species. Alternatively, they will be comparatively robust candidates for assisted migration, should it be required. The extraordinary success of white rhinoceros (*Ceratotherium simum var. simum*) reintroduction and recovery [30], might indicate the generality of our findings, at least amongst rhinoceros. So long as the anthropogenic causes of decline are treated (i.e., illegal hunting [31]), black rhinoceros recovery by reintroduction and restocking, and even assisted migration, should be comparatively easy. Groups of black rhinoceros of different size and composition can be moved successfully between different ecological contexts, and released into reserves that might already be stocked, and have poor habitat, so long as young are not used to restock populations and small cohorts are not reintroduced into the poorest habitats.

Our findings give confidence to the design of grand artificial meta-populations of similar conservation-reliant species that will require the translocation of individuals for assisted migration, reintroduction, and the genetic and demographic rescue of small populations by restocking. Achieving successful translocations of species like black rhinoceros, i.e., large asocial and biogeographically spread herbivores which are not predators and rarely prey, might not be nearly as socio-ecologically complicated as the literature leads us to believe. Such species will be robust to ecological challenge and resilient candidates for crisis management in a changing world.

## Materials and Methods

Reports on properties with populations of black rhinoceros in Namibia and South Africa 1981–2005 [17] were consulted for translocations and post-release survival of individually identifiable rhinos of known sex- and age-class. Data from the three sub-species in the region (i.e., *D.b.* var. *micheali* n = 43, *minor* n = 338, *bicornis* n = 301) were pooled. Information from those reports was supplemented with estimates of each reserve's relative carrying capacity (i.e., 0.015 to 0.884 rhino.km^−2^). Estimates of relative carrying capacity were derived from a regression model and sampling from 24 reserves in Kenya, Namibia and South Africa by Adcock *et al.* that are described in detail elsewhere [32], [33]. Briefly, the regression model included indices representing each reserve's black rhinoceros browse standing crop (percentage volume of selected woody and forb plant leaves, twigs and small branches within the 0 to 2 m feeding height range of black rhinoceros), potential rainfall- and temperature-dependent browse growth (monthly rainfall and minimum mid-winter, July, temperatures), soil fertility and fire regimes.

Reintroduction events are attempts to establish a population in an area once part of the species range but from which it became extinct. Restocking events are attempts to add individuals to an existing population of conspecifics within the species range [5]. Subsequent releases into the same reserve were classified as restocking events if they occurred more than one month after the first release because black rhinoceros appear to have developed home ranges within 30 days post-release [24]. For each translocated individual we compiled 40 individual rhino, release cohort, or reserve characteristic predictors for survival to one year after release [19].

Over- and under-fitting are a problem in multivariate analyses for detecting important predictors or combinations of predictors, especially where variables interact. This is particularly problematic for regression when the variables are gleaned from pre-existing databases because some important variables may not have been measured or included. For this reason we adopted an Information-Theoretic approach to testing hypotheses about the causes of mortality after release [13], [15] by constructing and comparing candidate models as hypotheses for translocation success.

To compile our candidate models we began with the leading models in our previous analysis [19] and appended a further suite of models describing interactions amongst predictors. The few leading models in our previous analysis for restocking success shared age class [34] in common and age was only found in the leading models. Age was also the only predictor with a credible interval (Bayesian measure of uncertainty, analogous to confidence intervals) that did not include zero and an effect size larger than any other predictor by a factor of two to three. Nevertheless, the possibility remains that the interaction of a number of other variables, particularly the quality of habitat, number or density of residents and post-release density, especially of bulls, exacerbates the vulnerability of young. So, to compile candidate models for our new analysis, we began with the leading models from our previous analysis and appended a further suite of models describing interactions amongst predictors.

The outcomes of previous analyses for reintroduction were ambiguous compared to those for restocking. Most models received similar support and no single hypothesis dominated [19]. Nevertheless, the coefficients of some variables and their effect sizes led us to speculate about their relative importance. In particular, the proportion of bulls and calves in the release cohort and quality of the habitat appeared to have greatest influence. We also speculated about the interaction between cohort size and the contributions of bulls and calves to the release cohort since large cohorts with calves, and small cohorts (one to three individuals) consisting entirely of bulls, had disproportionately high mortality rates. Thus, it is possible that the performance of models testing interactions amongst cohort size, the contribution of bulls and calves to the cohort and habitat quality might show improvement over leading models. In particular, we modeled two- and three-way interactions between cohort size and metrics of cohort composition, especially the contribution of bulls and calves, metrics of habitat quality and post-release population density, and individual age (i.e., vulnerability).

We centered continuous predictors by subtracting the mean and dividing by two standard deviations [14] and left binary predictors unmodified. We conducted all logistic regressions using *lme4* package in R 2.11.1 (R version 2.11.1, 2010-05-31, Copyright (C) 2010 The R Foundation for Statistical Computing) and fitted general linear mixed-models using Laplace approximations of maximum likelihood to calculate Akaike Information Criterion (AIC) for each model [35]. We used AIC because we were interested primarily in the influence of fixed-effects and the random effects structure remained constant among models. We calculated a second-order Akaike Information Criterion (AIC_c_) as our Information-Theoretic statistic because the number of structural parameters in models (*K*) was large relative to the number of contributing rhinoceros (n), particularly for the restocking dataset where n/K<40 [13]. We judged the relative power of candidate models by comparing their AIC_c_ and ratios of Akaike weights (*w_i_*). Models with lowest AIC_c_ have most support from the data. Relative support between candidate models was the difference between each model's AIC_c_ and the minimum value (AIC_c, min_) from all models (ΔAIC_c_). We considered models with ΔAIC_c_≤2 to have compelling support from the data and models with ΔAIC_c_>10 to have no support [13]. We included a base model including random effects, but without fixed effects, in the candidate set of models for comparison because we wanted to understand the amount of information in the data not explained by current theory. Models which performed worse than the base model could also be considered to be unsupported.
